# Role of practice standardization in outcome optimization for CDH

**DOI:** 10.1136/wjps-2024-000783

**Published:** 2024-03-21

**Authors:** Alexandra Dimmer, Robert Baird, Pramod Puligandla

**Affiliations:** 1 Harvey E. Beardmore Division of Pediatric Surgery, Department of Pediatric Surgery, Montreal Children's Hospital, Montreal, Quebec, Canada; 2 Division of Pediatric General and Thoracic Surgery, British Columbia Children's Hospital, Vancouver, British Columbia, Canada; 3 Harvey E. Beardmore Department of Pediatric Surgery, McGill University, Montreal, Quebec, Canada

**Keywords:** Evidence-Based Medicine, Health services research, Congenital Abnormalities, Standarization of care, Pediatric surgery, Clinical practice guideines

## Abstract

Standardization of care seeks to improve patient outcomes and healthcare delivery by reducing unwanted variations in care as well as promoting the efficient and effective use of healthcare resources. There are many types of standardization, with clinical practice guidelines (CPGs), based on a stringent assessment of evidence and expert consensus, being the hallmark of high-quality care. This article outlines the history of CPGs, their benefits and shortcomings, with a specific focus on standardization efforts as it relates to congenital diaphragmatic hernia management.

## Introduction

Standardization of work was historically designed to reduce unwanted variation in workflow, to improve efficiency, to reduce costs and to improve safety. In non-healthcare industries, such as automobile manufacturing, it has been used for decades as a means to proactively mitigate risk and to provide surveillance while implementing design features to prevent errors and minimize harm to workers. Despite being a more recent phenomenon, standardization in healthcare has similar aims: to reduce unwanted variations in care, to reduce costs and to improve healthcare delivery and outcomes, creating both quality and value in healthcare.^1^ The reasons for the variation observed in healthcare delivery today are multifaceted. Historically, medicine has operated on an apprenticeship model, whereby ‘best practices’ were handed down from generation to generation by mentors and experts in the field. This was often highly influenced by where physicians were trained and the reputation of their mentors.^2^ Indeed, personal practice was largely influenced by anecdotal evidence, and the patient care experience varied vastly between institutions and even within a single institution. Over the past few decades, there has been a growing body of literature challenging the model of apprenticeship training, and placing greater emphasis on practice patterns that are informed by best evidence. The exponential growth of medical research has made it difficult for individual practitioners to critically appraise the abundance of newly emerging evidence in a given field and to incorporate best practices. To address this problem, clinical tools have become an essential aid in decision-making as they distill evidence into practice recommendations. Furthermore, clinical decision aids have prompted the standardization of care for several specific conditions. In this article, we will address the potential benefits and hazards associated with care standardization and outline the specific efforts related to the standardization of care in congenital diaphragmatic hernia (CDH).

## The role of standardization in healthcare

### Introduction of clinical practice guidelines

**Figure 1:**
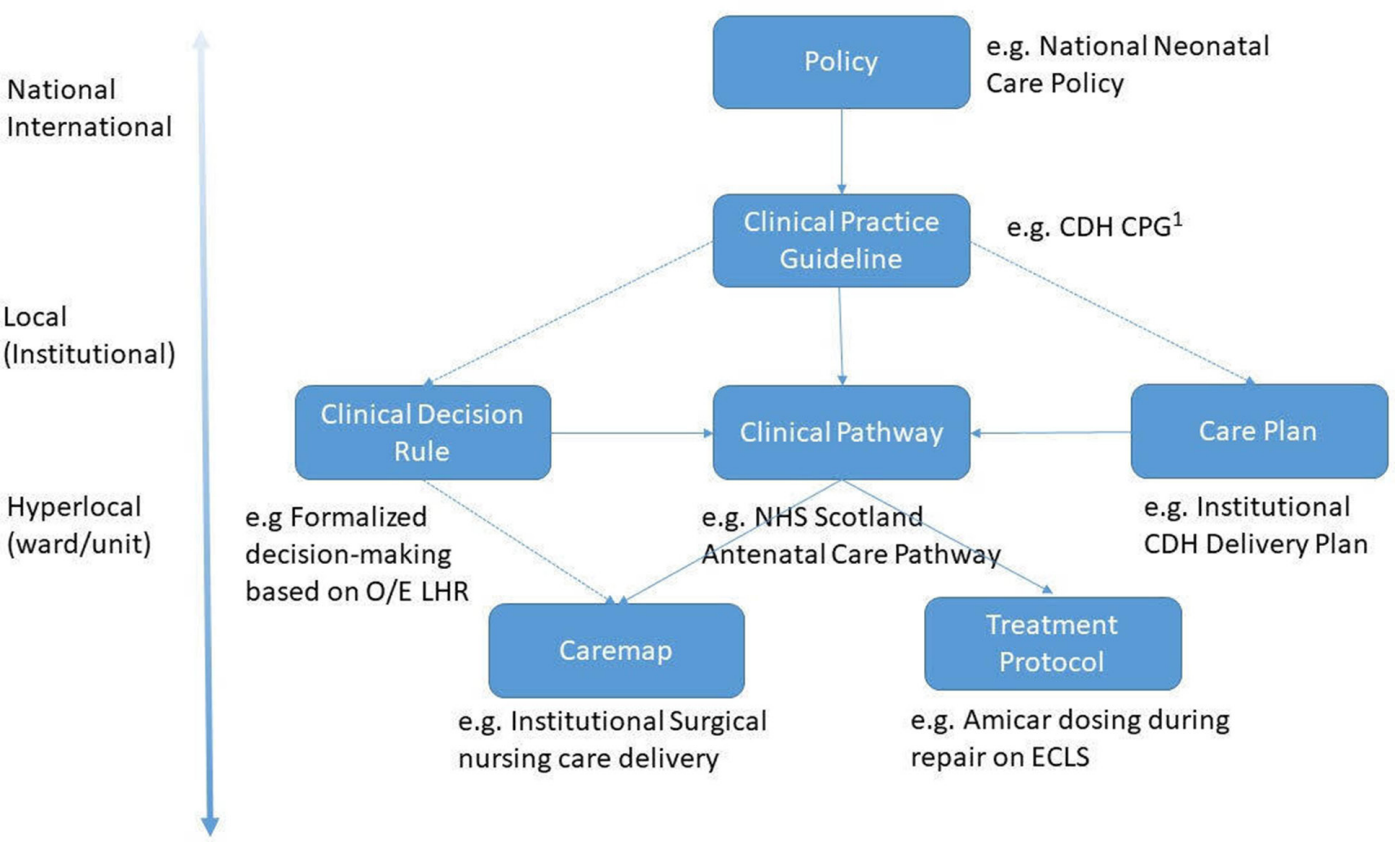
Hierarchical structure of standardization. CDH, congenital diaphragmatic hernia; NHS, National Health Service; LHR, luing-head ratio; O/E, observed-to-expected; CPG, clinical practice guideline; ECLS, extracorporeal life support.

Clinical standardization is the establishment of standards and protocols for caregivers to follow when treating patients. Its goals are to reduce unnecessary cost, to avoid unwarranted variation in treatment and to improve patient care and caregiver accountability^3^ (table 1). Standardization enables the delivery of reliable, high-quality care since it can be both measured and reproduced. Moreover, standardization assists clinicians in complex decision-making and offers value to the system. A common form of standardization in healthcare is the development and implementation of clinical practice guidelines (CPG), which use the current best evidence to inform decision-making.^4^ It is one of several examples of clinical care process specifications that include pathways, protocols, decision rules and care maps (table 1)—each with their respective application, aims and contextual domains. An example of the interplay between these processes is provided in figure 1, based on the hierarchal structure proposed by McLachlan *et al.* 5

**Table 1:** Common definitions

Term	Definition
Evidence-based medicine (EBM)	The conscientious, explicit and judicious use of current best evidence in making decisions about the care of individual patients.^66^
Clinical practice guidelines (CPGs)	Statements that include recommendations intended to optimize patient care that are informed by a systematic review of evidence and an assessment of the benefits and harms of alternative care options.^6^
Clinical pathway (CPW)	A structured, multidisciplinary care plan (‘inventory of action’) that translates guidelines or evidence into local practices and standardizes care for a specific population.^67^
Clinical care plan (CCP)	An organized, multidisciplinary day-by-day list of care activities with intermediate outcome-based goals that healthcare providers will undertake to support identified patient problems.^5^
Clinical decision rule (CDR)	Operationalization of an efficient approach to assessing probabilities for diagnostic, treatment and prognostic decisions and provide a link between published and clinical evidence, best practice and the diagnosis or clinical outcome under consideration.^5^
Clinical treatment protocol (CTP)	Clinical care activities developed on the basis of guideline-based evidence, and usually found incorporated into clinical pathways and described against a timeline.^5^

The history of CPGs in North America dates back to 1970, when the Institute of Medicine (IOM) was established as an independent, non-profit organization tasked with providing unbiased advice to healthcare decision-makers and the US public. In 1989, the IOM created the Agency for Health Care Policy and Research (AHCPR), which focused on healthcare outcomes and effectiveness research due to a growing concern regarding escalating healthcare costs, variations in practice patterns and ineffective healthcare services.^6^ As part of its mandate, the AHCPR created and updated guidelines to advise the medical public and to promote more consistent and efficient medical care across the USA. Together, the AHCPR worked with the IOM to determine best practices for medical guideline development. In 1990, the IOM defined CPGs as ‘systematically developed statements to assist practitioner and patient decisions about appropriate healthcare for specific clinical circumstances.’^6^ That definition has changed over time to place greater emphasis on the quality of evidence used to inform CPGs, highlighting the importance of systematic reviews as the gold standard for evaluating the effectiveness of medical treatments.^3^ The IOM now defines CPGs as ‘statements that include recommendations intended to optimize patient care that are informed by a systematic review of evidence and an assessment of the benefits and harms of alternative care options’^6^ (table 1).

CPGs have become essential decision-making tools for clinicians.^7^ CPGs are based on an exhaustive, systematic review of all available literature for a given topic, which is often supplemented with consensus opinion from a knowledgeable multidisciplinary expert panel.^6 8^ CPGs generally follow rigorous methodology and transparency to minimize biases and conflicts of interest and should be reviewed and revised with the emergence of new evidence, as needed.^6^ Well-designed CPGs have the potential to reduce unwanted variations in practice and improve healthcare delivery, quality and efficiency. Additionally, CPGs provide a basis for measuring provider/institutional performance (e.g., compliance with guideline statements and resultant patient outcomes) and subsequent quality improvement initiatives.^6^

### Examples of standardized healthcare in pediatric surgery

The value of standardization in healthcare is being increasingly recognized, particularly as it often leads to quality improvement initiatives. There are many clinical examples demonstrating outcome improvement after care standardization in pediatric surgery (table 2). One such example involves the management of pediatric perforated appendicitis. Despite being the most common acute surgical condition treated by pediatric surgeons,^9^ wide practice variations in pediatric appendicitis remain, potentially impacting patient outcomes.^10–12^ In response to an increased postoperative abscess rate at their institution, Yousef *et al.* revised an existing institutional pathway for perforated appendicitis by implementing standard antibiotic utilization, stratification of disease severity, standardization of the operative procedure, and refinement of discharge criteria.^13^ Prospective evaluation of 122 children treated for 20 months with the new standardized protocol compared with a retrospective cohort treated prior to standardization revealed a significant reduction in postoperative abscess and length of hospital stay.^13^

**Table 2:** Examples of care standardization in pediatric surgery

Context	Standardization	Level of evidence (LOE)	Outcome improvement	Reference
Childhood				
Perforated appendicitis	Grading scale, protocolized antibiotics	Prospective single center	Decreased length of stay (LOS)	Yousef *et al.* 13
Elective colon surgery	Operative care bundle	Prospective multi-center	Surgical site infection (SSI) rate	Tobias *et al.* 14
Elective inguinal surgery	Standardization of instrument tray	Prospective single center	Improved sterilization time	Koyle *et al.* 68
Gastrostomy	Gastrostomy tube care bundle	Prospective single center	Decreased gastrostomy tube dislodgement	Ruffolo *et al.* 69
Neonatal				
NEC	Standardized feeding regimen	Systematic review (SR)	Decreased incidence	Jasani *et al.* 70
Gastroschisis	Closure bundle	Retrospective single center	Less ventilator days	Haddock *et al.* 71
Gastroschisis	Feeding bundle	Systematic review (SR)	Quicker feeding, decreased mortality	Raduma *et al.* 72

NEC, necrotizing enterocolitis.

Another example of standardization in pediatric surgery involves pediatric colorectal surgery. Surgical site infection (SSI) is a source of significant morbidity in children undergoing colorectal surgery and results in increased healthcare resource utilization.^14 15^ Despite objective reductions in SSI rates in adults^16 17^ and application in children,^18–20^ the implementation of standardized preoperative care bundles remains widely variable with ongoing debate regarding their utility in reducing pediatric SSIs.^15 21^ To demonstrate the role that standardization could play in reducing SSIs, Tobias *et al.* performed a multicenter prospective cohort study evaluating the effect of an eight-element perioperative ‘colon bundle’ in reducing SSIs at 10 children’s hospitals.^14^ Patients were divided into low (1–4 elements) or high (5–8 elements) compliance cohorts based on bundle adherence. Superficial SSI within 30 days of surgery was significantly reduced among the high compliance cohort,^14^ underscoring the value of standardization in reducing morbidity in this patient population.

### Benefits of CPGs

CPGs offer several benefits to patients, healthcare professionals and policymakers alike. For patients, the most significant benefit is the potential to reduce mortality and morbidity by promoting treatments with proven benefits, and discouraging ineffective or harmful treatments.^22^ Furthermore, CPGs promote consistency in medical care, helping to ensure that patients receive the same treatment regardless of their geographical location or clinician expertise/interests.^22^ CPGs have the ability to influence public policy and advocate for equitable distribution of healthcare resources. Guidelines also have the potential to raise awareness of under-recognized health problems and/or effective treatments, with the consequence of increasing the availability of services that were not previously offered based on evidence promoting their efficacy.^22^ For healthcare professionals, CPGs have the ability to improve the quality of healthcare decision-making by distilling the large amount of available evidence into explicit care recommendations. These recommendations are often graded based on the quality of evidence informing them (e.g., Grading of Recommendations, Assessment, Development and Evaluation (GRADE)^23^), and thereby highlight practices that may be ineffective, dangerous or wasteful of limited healthcare resources.^22 24^ As mentioned previously, CPGs also support quality improvement initiatives either through compliance measurements with established care practices or through more meaningful measurement and study of care processes needed to meet care standards.^22^ Finally, CPGs offer benefits to policymakers and healthcare systems by improving efficiency, and reducing costs—a benefit which is often seen as a primary driving force for the development of CPGs in private healthcare systems such as the USA.^22^ In their study of the effect of standardization on reducing costs for pediatric patients, Friedman and Fulton found that standardized assessment and management plans, similar to CPGs, had the potential to reduce patient care costs in pediatric cardiology by up to 51%, when implemented consistently.^25^

### Potential weaknesses of CPG

Despite their many benefits, CPGs have inherent limitations and potential harms. First, and most important, the recommendations contained within CPGs may be wrong. While guidelines should be based on a rigorous systematic review of available evidence, misinterpretation of data or the failure to address biases and flaws in study design may result in poorly designed or even harmful recommendations.^22 24^ Additionally, CPGs may become irrelevant, if they do not reflect ongoing advancement and innovation in the field. Without regular appraisal of new or emerging evidence, and the subsequent updating of CPG recommendations, providers may base their decision-making on outdated evidence, which may contribute to poorer outcomes, or in unnecessary healthcare resource utilization.^25^

CPGs may also lead to inflexible patient care, which does not account for individualized patient needs or resource considerations.^7 26^ Indeed, guidelines rarely consider studies on the social determinants of health and their impact on patient care delivery.^7^ In their review of the non-clinical influences that affect clinical decision-making, Hajjaj *et al.* found that the failure of CPGs to recognize factors such as socioeconomic status, quality of life and patient expectations was the greatest barrier to their widespread implementation in everyday practice.^27^ CPGs rarely account for the difference in resources available at individual institutions and almost never account for a resource–poor environment. Given the financial burden associated with the de novo development of CPGs, they rarely emanate from low-to-middle income countries, requiring these countries to adopt, contextualize or adapt existing CPGs from high-income countries to fit their local context or resource constraints.^28^

Additionally, CPGs have the potential to harm a medical professional’s autonomy in two distinct ways: first, by allowing for the transfer of protocolized work to less skilled professionals or surrogates, or to the patients themselves^7^; and second, by providing a means with which to monitor, audit and compare healthcare professionals’ work, possibly allowing for legal proceedings against physicians.^7 24^ Compliance with the recommendations outlined in CPGs is not a sufficient defense against the possible liability of medical negligence during medicolegal proceedings. However widely followed or well-composed, CPGs may be seen as the de facto ‘standard of care’ – and failure to comply with these recommendations in the setting of patient harm, risks litigation against healthcare providers.^29 30^ Within private medical systems, insurance providers may use CPGs as the basis with which to approve or deny medical care, without understanding the individualities of each patient which may require deviation from guideline standards.^24 31^ This inability to see beyond CPGs has the potential to hinder physician decision-making autonomy and can have dire consequences on patient care.

## Standardization in congenital diaphragmatic hernia

### Rationale

Over the past several decades, survival in CDH has improved to nearly 80%,^32^ largely due to advancements in neonatal intensive care and a greater understanding of the underlying pathophysiology of CDH.^33^ Despite the improvement in survival over the past three decades, mortality rates have plateaued over the last several years, prompting the question of missed opportunities to further improve not only mortality but also the subsequent morbidity of CDH infants.

CDH management is complex, requiring highly specialized interdisciplinary teams during three unique phases of life: prenatal, perinatal and posthospital discharge. Hospitals which manage CDH must have expertise in neonatal intensive care, advanced cardiopulmonary support capabilities, including extracorporeal life support (ECLS) and the management of severe pulmonary hypertension, as well as expert teams capable of repairing the diaphragmatic defect. Importantly, the management of CDH infants remains interdisciplinary across the patient lifespan and encompasses a team that should include neonatologists, gastroenterologists, respirologists, pediatric anesthesiologists, pediatric surgeons, developmental pediatricians and physical and occupational therapists.^34^ With many treating specialists, and a variety of unique practice patterns, CDH management is highly variable across treating institutions,^35^ especially with regards to the use of ventilation strategies, antenatal steroids, approach to treatment of pulmonary hypertension, the use of ECLS and the timing and approach to surgical repair.^36 37^

A significant barrier to improving CDH outcomes has been the lack of high-quality evidence to inform best practice, and the high variability in practice patterns among treating centers.^38^ A systematic review performed by the American Pediatric Surgical Association Outcomes and Evidence-based Practice Committee aimed at providing best practice recommendations for specific aspects of CDH management, identified a lack of high-quality evidence, which prevented the identification of best practices.^32^ The authors also found that even when adequate evidence was available, practice pattern variation remained, despite evidence demonstrating a lack of benefit of specific therapies.^32^ Further confounding standardization efforts is the relative rarity of CDH, which occurs in roughly 1 in every 3500 live births.^33^ As such, single center experiences may not be representative of best practice,^35^ highlighting the need for multi-institutional or population-based guidelines that can leverage a much broader CDH experience.^39^

Jancelewicz *et al.* identified significant heterogeneity among CPGs utilized for CDH management in multiple institutions across North America.^38^ In this study, North American members of the Congenital Diaphragmatic Hernia Study Group (CDHSG) and Pediatric Surgical Research Collaborative were contacted to ascertain if they had an institutional CPG for CDH management, to determine its contents, and to confirm if they offered ECLS at their institution. The authors found that at least one-third of surveyed centers *did not* have a CPG. While their analysis also highlighted significant content variability among those centres that did have a CPG,^38^ there was general alignment with respect to specific care elements, most notably ventilatory management, aspects of postnatal resuscitation and the criteria for transitions in care.^38^ This study was the first to assess practice patterns across North America, highlighting the inconsistency of existing CDH guidelines, while simultaneously exposing opportunities for improved alignment.

### Economic considerations

Given the severity of disease and the multidisciplinary nature of CDH management, it is no surprise that CDH remains one of the most costly congenital conditions.^35 40^ A single-institutional study exploring the cost of inpatient perinatal care for a cohort of CDH infants found that the average cost per survivor was more than threefold higher for a CDH infant compared with an illness-severity matched cohort of NICU patients in the same Canadian institution.^41^ The average cost per patient for initial CDH hospitalization in the USA is estimated to be greater than $350 000. The extrapolated cost for the care of CDH infants from birth to hospital discharge in the USA exceeds $390 million annually, making CDH the pediatric surgical condition with the highest median costs in the USA.^42^ The economic burden on the healthcare system is further increased in infants with severe CDH who require ECLS, as ECLS use has been shown to increase costs by 2.5–3.5-fold when compared with CDH infants not requiring ECLS.^42^ Despite an understanding of the cost of initial hospitalization for these infants, to date, no study has estimated the costs of CDH survival beyond initial discharge.^43^ Given the significant postdischarge morbidity associated with CDH, it is certain that the lifetime direct and indirect costs to the healthcare system and to CDH patients’ families are substantial, underscoring further the need to leverage standardization for fiscal benefit.^25 44^

### Examples of standardization in CDH

Over the last 15 years, there have been notable efforts to standardize care in CDH. An initial CDH Study Group (CDHSG) study in 2007 used prospectively collected registry data from 1995 to 2004 to identify the correlation of defect size (determined intraoperatively) with mortality, and how defect size was a likely surrogate for the degree of pulmonary hypoplasia seen in CDH infants.^45^ The greatest criticism of this finding was the limited accuracy of defect size, due in part to a lack of standardized intraoperative reporting across institutions. In response to this criticism, the CDH Study Group created a standard classification system based on the degree of diaphragmatic muscle found intraoperatively.^46^ Using this standardized reporting system, Lally *et al.* demonstrated that defect size and the presence of severe cardiac anomalies were most strongly associated with poor CDH outcome.^35^

In 2010, the CDH EURO Consortium, a collaboration of CDH centers in Western Europe, published a CPG for the postnatal management of CDH based on a systematic review of best evidence combined with expert opinion. The CPG was created during a consensus meeting among participating sites, each of which cared for at least 10 CDH infants annually. The level of evidence after literature review was graded using the Scottish Intercollegiate Guidelines Network Criteria.^47^ Differences in opinion were resolved by five individual experts until full consensus was reached. The final CDH EURO Consortium consensus statement encompassing 36 recommendations represented the opinion of all consortium members.^39^ Given that no multicenter randomized control trials were used to build these recommendations, the authors cautioned that their CPG only represented a consensus guideline, and not a best practice document, due to a lack of available evidence.^39^ In a subsequent multicenter study assessing the impact of this CPG to influence patient outcomes, the authors reported a mortality reduction from 33% to 12% (p=0.004) after implementation of the guidelines, with no impact on ECLS utilization, or prevalence of secondary pulmonary morbidity, such as bronchopulmonary dysplasia.^48^ The results of this study underscored the value of standardization and also revealed the need for multi-center prospective studies to further inform best practice in CDH.

The paucity of high-level evidence to inform CDH care recommendations, as highlighted by the CDH Euro consortium consensus statement, was a call for more prospective study in CDH. The VICI trial—which sought to assess conventional mechanical ventilation versus high-frequency oscillatory ventilation as the initial ventilation strategy in CDH infants^49^ is one of the few randomized trials in CDH and was a direct consequence of standardization efforts. As a prerequisite for participation in this trial, sites had to adopt the standardized EURO Consortium care recommendations. Five years after publication of the original guidelines, the number of participating institutions in the CDH EURO consortium increased from 13 to 22 centers, and the guidelines were updated to reflect advances in CDH care since its original publication.^50^

At the time that the initial CDH Euro consortium consensus statement was published, the Canadian Pediatric Surgery Network (CAPSNet) undertook a population-based study to assess management and outcome data for all infants born with CDH over a 4-year period across the 16 tertiary-level perinatal centers across Canada. The purpose of the study was to highlight interinstitutional variability in CDH treatment and its effect on outcomes, including mortality, length of stay and duration of ventilation; the study would also form the basis for the development of future national quality improvement initiatives.^37^ While the overall CAPSNet CDH survival was over 80%, significant interinstitutional variability existed, with survival ranging between 40–100%.^37^ Additionally, significant interinstitutional variation was present with regards to obstetrical management, mode of ventilation, use of muscle relaxants and the timing and type of surgical closure.^37^ Given the significant variation of care observed between Canadian centers, the Canadian CDH Collaborative (CCC) was formed in 2015 with the intent to create an ‘evidence-based and consensus-driven national guideline for the health surveillance and care of patients with CDH from prenatal diagnosis through to long-term follow-up.’^51^ The CCC is composed of a panel of geographically representative specialists across Canada with diverse expertise in neonatology, pediatric surgery, pediatric anesthesia, maternal-fetal medicine, pediatric critical care and pediatric cardiology. The panel was divided into working groups who appraised existing evidence using GRADE methodology.^23^ The evidence review focused on a set of 14 topics across the phases of CDH care (prenatal, in-hospital and postdischarge); care recommendations and evidence summaries informing those recommendations were subsequently drafted. These recommendations and evidence summaries were reviewed during a 2-day in-person meeting, with consensus established through live, electronic voting using a modified Delphi technique. The guidelines were subsequently published and distributed to professional societies across Canada.^51^ In comparison with the EURO Consortium guidelines, the Canadian guidelines were the first to address the role of experimental therapies and also to offer recommendations regarding long-term surveillance of CDH survivors.^51^ Formal comment on long-term health surveillance was an important component of the CCC CPG given that greater than 50% of CDH survivors experienced some form of long-term multisystem morbidity that affected neurodevelopment, growth and nutrition as well as the cardiac, gastrointestinal and musculoskeletal systems.^33 52–54^ Like the EURO Consortium guidelines, the Canadian guidelines have also undergone a recent evergreening process. This update includes 15 new CDH care recommendations, including recommendations for pain management, analgesia and neuromuscular blockade, and 20 revisions to the existing recommendations.^34^

The same year, the CCC was established, the American Heart Association (AHA) and American Thoracic Society published the first set of guidelines for the diagnosis, evaluation and management of pediatric pulmonary hypertension.^55^ Unlike in the adult population where several guidelines and treatment options existed, there once again remained a paucity of high-quality studies which addressed pediatric pulmonary hypertension and its treatment options. The recommendations within these guidelines reflect the state of existing literature, along with the consensus of expert opinion to account for the lack of high-quality randomized trials in the field.^55^ Given that pulmonary hypertension remains a significant cause for the mortality and morbidity seen in CDH infants, these guidelines are a welcome clinical decision-aid for those providers treating infants with CDH. The AHA guidelines, along with the guidelines for ECLS management in CDH infants, put forth by the Extracorporeal Life Support Organization in 2021,^56^ informed care recommendations in the 2023 update of the CCC guidelines.

### Barriers to implementation of standardization in CDH

Although standardization has the potential to result in outcome improvement and cost reduction, the complexity of CDH care, including the need for an interdisciplinary approach with multiple treating providers, presents a significant challenge to standardization.^38^ While standardization of care has been shown to be of benefit in CDH,^57 58^ many CPGs are not widely implemented, suggesting important barriers which must be considered. A recent scoping review sought to determine the most important barriers to guideline implementation.^59^ Barriers related to physician knowledge included lack of awareness of guidelines as well as a lack of familiarity with the guidelines and its recommendations. Additional barriers included a lack of agreement with guideline recommendations, a lack of evidence, the plausibility of recommendations, the complexity of the guidelines, a lack of access to the guidelines, a lack of applicability and a lack of local resources.^59^ Another recent study employed a survey sent to physicians from many specialties to assess barriers to guideline implementation. Three major barriers to guideline adherence were identified and included guideline complexity, weak or conditional guideline recommendations and clinical time constraints.^60^ The success of CPGs rests on identifying barriers to implementation and developing strategies to overcome them.^59^

Following the publication of the CCC CPGs in 2018, a survey was sent to key stakeholders (surgical, nursing, medical, other health professionals) at each of the 18 participating CAPSNet institutions to assess the readiness for change and implementation of the new CDH care recommendations. The readiness for change focused on three key domains: the perception of strength of evidence, the quality of the context or environment for guideline implementation and local facilitation.^61^ More than 75% of respondents were aware of the guidelines, with greater than 60% utilizing the entire guideline, and another one quarter utilizing certain sections.^61^ Despite awareness of the guidelines by the majority of respondents, several barriers to universal implementation were identified. Many respondents cited lack of resources, including both human and financial resources as barriers to change. Additionally, greater than 40% of participants felt that further feedback regarding CDH outcomes and patient measures, as well as data on the effects of clinical decision-making, was necessary to effect change. Despite these limitations, the majority of respondents felt that greater than 75% of the guideline recommendations were implementable at their institution with adequate support and resources.^61^ The results of this study demonstrate the importance of assessing the practice landscape of clinical guidelines to determine barriers to their implementation. Although barriers exist, they can often be remedied with additional resource support.

To overcome these barriers to implementation, stakeholders may consider dissemination tools to increase the knowledge of CPGs and facilitate easier access. As part of a modern uptake and implementation strategy following the publication of the CCC guidelines, a smartphone app was developed to allow for ‘fingertip access’ to the guidelines, with the ability to easily update recommendations as new evidence became available.^62^ In addition to the recommendations published in the CPG, the app offers additional support features and resources, such as neonatal intensive care rounding checklists and the American Association of Pediatrics long-term CDH surveillance schedule,^63^ in addition to links to risk stratification calculators. Finally, a risk calculator was also created for the CDH Study Group clinical prediction rule, stratifying infants into mortality risk categories based on prenatal indices.^62^ In response to readiness survey comments regarding the need for regular compliance assessments to ensure widespread guideline uptake and utilization,^61^ a quality improvement tool for tracking institutional compliance with the guidelines was designed and included as a resource within the mobile app.^62^ The app has been used globally, with the majority of users in Canada, followed by the USA, and subsequently Brazil.

### Future directions

Standardization in CDH must continue to address ongoing advances in research and innovation. There is also a need to demonstrate the effect of CPGs on improving CDH outcomes. This may be best accomplished using large multicenter studies to compare preguideline and postguideline implementation clinical outcomes and compliance with identified best practices. This is an important feedback mechanism to ensure ongoing adherence to care recommendations as well as to identify potential new areas of care innovation. Recent guideline update efforts have also identified many areas in dire need of ongoing prospective evaluation. The paucity of high-level evidence remains as a potential obstacle to broader adoption of existing CPGs or to potential bias due to reliance on expert consensus rather than high level evidence. In addition, disparities in healthcare access and delivery remain a significant concern. Future guideline development should consider health equity, with the goal of addressing these disparities to provide equitable care for all patients, an important aspect of high-quality healthcare. Equally important will be the identification and incorporation of patient-oriented outcomes and experiences as an untapped opportunity for quality improvement. There is also a potential role for patient partners in the development process, especially when it comes to identifying outcome goals, and priority areas for standardization.

## Conclusion

The IOM defines six dimensions of healthcare quality: safety, effective, timely, efficient, patient-centered and equitable.^64^ The standardization of healthcare delivery addresses several of these domains to various degrees—most notably safety, efficacy and efficiency. Future efforts aimed at addressing patient-centered care and equitable access and delivery will further improve healthcare quality. Given that CDH is a high risk, low volume condition, prioritization of structural improvement metrics is likely to have the greatest impact on overall quality of care delivery.^65^ As demonstrated by the many examples in this review, CPGs have the ability to standardize and ultimately improve the quality of care delivery for patients with CDH across institutions. Given the paucity of high-quality randomized trials, standardized care can help to facilitate multi-institutional studies, which will further strengthen the recommendations offered by CPGs. Despite many benefits of standardization, clinicians should be wary of possible limitations, including generalizability of recommendations to their practice settings, and individual patients. CPGs require frequent updates with the emergence of new literature, and future research should address the optimal frequency with which these guidelines should be updated. In addition, further research is necessary to address both the economic impact of standardization of care in CDH and the possible impact on clinical outcomes.
